# Outcomes of liver–kidney transplantation in patients with primary hyperoxaluria: an analysis of the scientific registry of transplant recipients database

**DOI:** 10.1186/s12876-020-01349-1

**Published:** 2020-07-03

**Authors:** Jie Xiang, Zheng Chen, Fangshen Xu, Shengmin Mei, Zhiwei Li, Jie Zhou, Yinlei Dong, Yangjun Gu, Zhichao Huang, Zhenhua Hu

**Affiliations:** 1 Division of Hepatobiliary and Pancreatic Surgery, Department of Surgery, First Affiliated Hospital, School of Medicine, Key Laboratory of Combined Multi-Organ Transplantation, Ministry of Public Health Key Laboratory of Organ Transplantation, Zhejiang University, No.79 Qingchun Road, Hangzhou, 310003 Zhejiang Province China; 2 Division of Hepatobiliary and Pancreatic Surgery, Department of Surgery, Fourth Affiliated Hospital, School of Medicine, Zhejiang University, Yiwu, Zhejiang China; 3 Division of Hepatobiliary and Pancreatic Surgery, Yiwu Central Hospital, Yiwu, Zhejiang China

**Keywords:** Primary hyperoxaluria, Inherited disease, Liver–kidney transplantation, Combined liver and kidney transplantation, Sequential liver and kidney transplantation

## Abstract

**Background:**

Primary hyperoxaluria (PH) is an inherited disease lacking of hepatic oxalic acid metabolic enzymes which could lead to irreverisible renal damage. Currently, liver–kidney transplantation is a curative but highly invasive therapy used to treat patients with PH. However, limited studies have focused on combined liver–kidney transplantation (CLKT) and sequential liver and kidney transplantation (SLKT) in patients with PH.

**Methods:**

The present study included 201 patients with PH who received both liver and kidney transplants and who were listed on the Scientific Registry of Transplant Recipients from 1987 to 2018. According to the liver–kidney transplant procedure, patients were separated into a CLKT group and a SLKT group. Patient demographics and transplant outcomes were assessed in each group.

**Results:**

Compared with the SLKT group, The CLKT group got a worse pretransplant dialysis condition in both the proportion of patients under pretransplant dialysis (*p* = 0.048) and the duration of the pretransplant dialysis (*p* < 0.001). The SLKT group got higher human leukocyte antigen mismatch score of kidney donor (*p* < 0.001) and liver donor (*p* = 0.003). The CLKT group utilized higher proportion (98.9%) of organs from a single deceased donor, while the SLKT group utilized 75.0% of organs from deceased liver donors and only 35.0% of organs from deceased kidney donors (*p* < 0.001). Kidney function measured by serum creatinine concentration before liver transplantation (LT) or CLKT was similar (*p* = 0.305) between groups. Patient survival was not significantly different between the two groups (*p* = 0.717) and liver (*p* = 0.685) and kidney (*p* = 0.464) graft outcomes were comparable between the two groups.

**Conclusions:**

SLKT seems to be an alternative option with strict condition for CLKT, further exploration about the SLKT is still required.

## Background

Primary hyperoxaluria (PH) is a kind of congenital disorder that results from abnormal glyoxylate metabolism owing to deficiency of hepatic enzymes [1]. However, symptoms of PH are usually observed in the urinary tract, and are related to continuous oxalate deposition and irreversible renal damage resulting in end-stage kidney disease (ESKD). In patients with ESKD, a high level of oxalate production is compounded by a high daily dietary oxalate intake and impaired oxalate elimination, which causes a step-by-step increase in plasmatic oxalate and results in calcium oxalate crystals depositions in extra-renal tissues and organs, such as the skin, bones, and heart [2]. Systemic oxalosis is observed in the later stage of ESKD and can be fatal in patient with PH [3]. Patients with type 1 PH (PH1), which is induced by loss of hepatic alanine-glyoxylate aminotransferase [4] account for the majority of patients with PH worldwide [5, 6]. Since simple renal therapies such as dialysis or kidney transplant [1] are not able to solve oxalate accumulation, more effective strategies are under exploration.

Liver–kidney transplantation (LKT) is proposed because it could not only rescue hepatic enzyme deficiency, but it could also replace the dysfunctional kidney, especially in patients with PH1. Combined liver and kidney transplantation (CLKT) is the mainstream transplant strategy for LKT procedures, and global centers have confirmed the feasibility of the procedure with encouraging long-term results, even in pediatric patients [7, 8]. However, this procedure demands liver and kidney donors, usually a single deceased donor, which is costly and strict owing to organ shortages [9]. Sequential liver and kidney transplantation (SLKT) is an alternative strategy that separates the CLKT procedure into two steps: liver transplant (LT) and kidney transplant (KT). The application of intensive dialysis after LT could help the liver graft to mobilize and eliminate the general oxalate burden before KT, which may improve renal graft survival outcomes [10, 11]. However, previous studies pointed out the inferior patient survial in SLKT or CLKT compared with kidney transplant in children, and these studies may suggest the the overall high risk of multiorgan transplantation, even in sequential procedure [8, 12]. There were researchers believed that liver allograft provided immunoprotection for kidney graft only if both organs are transplanted simultaneously [13]. But recently, Kumiko et al. pointed out that the preceding liver allograft could contribute to the immunological protection, which may benefit the long term renal allograft outcome in patients after SLKT from a single donor when compared with kidney transplant alone [9]. However, the details of the SLKT in specific disease condition like PH, such as the donor-recipient match condition, the timings of LT and sequential KT, are still not reach a consensus.

Although different series and registry studies about PH have been published worldwide, most of them adopted a single-center design. The limited number of patients in these cohorts means that the results are not statistically significant. Little is known about multi-center research into CLKT and SLKT in patients with PH. Calinescu, A. M. et al. analyzed all primary pediatric CLKT cases in the Scientific Registry of Transplant Recipients (SRTR) and found that CLKT has similar survival outcomes when compared with isolated LT (including SLKT) and patients with PH present with worse outcomes than patients without PH [8]. The details in the group of patients with PH were not mentioned in this study. In this study, we sought to clarify the differences between CLKT and SLKT in patients with PH in the SRTR database to involve patients reported to date as much as possible.

## Methods

### Data source

This study was based on the SRTR, which includes data from all donors, wait-listed candidates, and transplant recipients in the U.S.A., submitted by the members of the Organ Procurement and Transplantation Network (OPTN). The Health Resources and Services Administration (HRSA), the U.S. Department of Health and Human Services, provides oversight into the activities of the OPTN and SRTR contractors. The whole study was reviewed and approved by the ethics committee at Zhejiang University, China (No.2019–1022-1).

### Study population

PH was defined using specific diagnosis codes (code 4307 in the LT database, code 3013 in the KT database) or indications of PH specified in the diagnosis text field. After data integration, only patients from 1987 to 2018 underwent CLKT or SLKT (with prior LT) according to the time interval between LT and KT. Patients who had received a multi-visceral transplant or who underwent re-transplantation were excluded from the study.

### Study outcomes

The primary outcomes were post-transplant patient survival, liver graft survival, and kidney graft survival. Patient survival was defined as the time from the initial transplant to patient death or final follow up. Liver graft survival was defined by the time interval between date of LT and liver graft failure date, patient death date, or final follow up date. Kidney graft survival was defined by the time interval from date of KT to kidney graft dysfunction date, patient death date, or final follow up date. Either liver or kidney graft dysfunction was defined by a clear graft failure record or re-transplantation date.

### Statistics

Demographic and clinical characteristics of recipients at initial transplant and liver and kidney donor and graft characteristics were described using median (interquartile range [IQR]) and frequency (percentages), respectively. Continuous and categorical variables of the analyzed groups were compared using the Mann–Whitney U test and chi-squared tests or Fisher’s exact test, as appropriate. A two-sided α level of < 0.05 indicated statistical significance. The Kaplan–Meier method was applied to estimate the probability of patient survival and graft survival, and the log-rank test was used to make between-group comparisons. Statistical analysis was performed using SPSS 22.0 (IBM Corp, Armonk, NY, U.S.A.).

## Results

According to our definition, a total of 201 patients from 1987 to 2018 were included in this study; 181 patients underwent CLKT, while 20 patients underwent SLKT.

The demographic and clinical features of recipients at initial transplantation are outlined in Table 1. We found that the pretransplant dialysis condition were significantly different between groups including pretransplant dialysis type and dialysis duration. The proportion of patients under dialysis (including hemodialysis and peritoneal dialysis) was higher in the CLKT group compared with the SLKT group (87.9% vs. 64.3%, *p* = 0.048), and the pretransplant dialysis duration was also found longer in the CLKT group than the SLKT group (17.0 months (IQR 7.0, 22.0) vs. 4.0 months (IQR 0.0, 12.3), *p* < 0.001). There were no significant differences between the two groups in terms of age (*p* = 0.183), body mass index (*p* = 0.133), gender (*p* = 0.651), race (*p* = 0.378), and serum creatinine concentration (*p* = 0.312). The median waiting time from the listing date to the initial transplant date was 5.0 (IQR 1.5, 9) in the CLKT group compared with 2 (IQR 1.0, 7.5) in the SLKT group (*p* = 0.094).

**Table 1:** Recipient characteristics at initial transplantation in CLKT and SLKT groups

Recipient characteristics	CLKT (***n*** = 181)	SLKT (***n*** = 20)	***P*** value
Median age, year (IQR)	20.0 (8.0, 31.5)	11.5 (1.85, 27.3)	0.183
BMI (median, IQR)	21.5 (18.0, 26.9)	18.8 (16.9, 23.1)	0.133
Gender			0.651
Male	99 (54.7%)	12 (60.0%)	
Female	82 (45.3%)	8 (40.0%)	
Race			0.378
White	135 (74.6%)	17 (85.0%)	
Black	12 (6.6%)	1 (5.0%)	
American Indian or Alaskan Native	2 (1.1%)	1 (5.0%)	
Others	1 (0.6%)	0 (0.0%)	
Asian	8 (4.4%)	0 (0.0%)	
Hispanic	23 (12.7%)	1 (5.0%)	
Serum creatinine (mg/dL, median, IQR)	5.1 (3.4, 7.2)	5.6 (2.8, 10.7)	0.312
Pretransplant dialysis type			0.048
No dialysis	11 (8.3%)	3 (21.4%)	
Hemodialysis	100 (75.8%)	6 (42.9%)	
Peritoneal Dialysis	16 (12.1%)	3 (21.4%)	
Dialysis Status Unknown	5 (3.8%)	2 (14.3%)	
Pretransplant dialysis duration (median, IQR)	17.0 (7.0,22.0)	4.0 (0.0,12.3)	< 0.001
Median waiting time from listing to transplant, months (IQR)	5.0 (1.5, 9)	2 (1.0, 7.5)	0.094

*CLKT* Combined liver and kidney transplant, *SLKT* Sequential liver and kidney transplant, *IQR* Interquartile range, *BMI* Body mass index, *HLA* Human leukocyte antigen

Donor and graft characteristics are summarized in Table 2. For liver donors, the CLKT group used a higher proportion of deceased donors (98.9%) when compared with the SLKT group (75.0%; *P* < 0.001). As for LT procedure type, the proportion of patients receiving whole liver graft was higher in the CLKT group compared with the SLKT group (80.1% vs. 60.0%; *P* = 0.025). Donor age, gender, race, and cause of death were similar between groups. In kidney donors, the donor age was higher in the CLKT group (median age = 20 years) compared with the SLKT group (median age = 34 years; *p* < 0.001). The CLKT group received organs from deceased donors in a greater percentage of cases (98.9%) compared with the SLKT group (35.0%; *p* < 0.001). Donor gender, race, serum creatinine concentration, and cause of death were similar between groups.

**Table 2:** Donor and graft characteristics in CLKT and SLKT groups

Donor and graft characteristics	CLKT (***n*** = 181)	SLKT (***n*** = 20)	***P*** value
**Liver**			
Median age, year (IQR)	20.0 (12.0, 30.5)	21.0 (10.0, 37.5)	0.977
Gender			0.492
Male	105 (58.0%)	10 (50.0%)	
Female	76 (42.0%)	10 (50.0%)	
Race			0.087
White	116 (64.1%)	19 (95.0%)	
Black	22 (12.2%)	1 (5.0%)	
American Indian or Alaskan Native	2 (1.1%)	0 (0.0%)	
Asian	4 (2.2%)	0 (0.0%)	
Hispanic	37 (20.4%)	0 (0.0%)	
Serum creatinine (mg/dL, median, IQR)	0.80 (0.60, 1.00)	0.71 (0.44, 0.90)	0.243
HLA mismatch			0.003
0	0 (0.0%)	1 (5.9%)	
1	2 (1.3%)	1 (5.9%)	
2	2 (1.3%)	2 (11.8%)	
3	13 (8.2%)	2 (11.8%)	
4	42 (26.6%)	4 (23.5%)	
5	62 (39.2%)	5 (29.4%)	
6	37 (23.4%)	2 (11.8%)	
Liver transplant procedure			0.025
Whole liver	145 (80.1%)	12 (60.0%)	
Partial liver	13 (7.2%)	5 (25.0%)	
Split liver	23 (12.7%)	3 (15%)	
Donor type			< 0.001
Deceased	179 (98.9%)	15 (75.0%)	
Living	2 (1.1%)	5 (25.0%)	
Donor cause of death			0.937
Anoxia	40 (22.3%)	3 (20.0%)	
Cerebrovascular/stroke	33 (18.4%)	3 (20.0%)	
Head trauma	96 (53.6%)	8 (53.3%)	
CNS tumor	4 (2.2%)	0 (0.0%)	
Other	6 (3.4%)	1 (6.7%)	
**Kidney**			
Median age, year (IQR)	20.0 (12.0, 31.0)	34.0 (28.0, 43.8)	< 0.001
Gender			0.287
Male	77 (42.5%)	11 (55.0%)	
Female	104 (57.5%)	9 (45.0%)	
Race			0.169
White	117 (64.6%)	18 (90.0%)	
Black	22 (12.2%)	2 (10.0%)	
American Indian or Alaskan Native	2 (1.1%)	0 (0.0%)	
Asian	4 (2.2%)	0 (0.0%)	
Hispanic	36 (19.9%)	0 (0.0%)	
Serum creatinine (mg/dL, median, IQR)	0.80 (0.60, 1.00)	1.00 (0.23, 1.55)	0.771
HLA mismatch			< 0.001
0	0 (0.0%)	2 (10.5%)	
1	2 (1.3%)	2 (10.5%)	
2	2 (1.3%)	2 (10.5%)	
3	13 (8.2%)	3 (15.8%)	
4	42 (26.4%)	6 (31.6%)	
5	63 (39.6%)	3 (15.8%)	
6	37 (23.3%)	1 (5.3%)	
Kidney transplant procedure			0.698
Left kidney	110 (60.8%)	13 (65.0%)	
Right kidney	65 (35.9%)	7 (35.0%)	
En bloc	6 (3.3%)	0 (0.0%)	
Donor type			< 0.001
Deceased	179 (98.9%)	7 (35.0%)	
Living	2 (1.1%)	13 (65.9%)	
Donor cause of death			0.622
Anoxia	40 (22.3%)	2 (28.6%)	
Cerebrovascular/stroke	33 (18.4%)	1 (14.3%)	
Head trauma	96 (53.6%)	3 (42.9%)	
CNS tumor	4 (2.2%)	0 (0.0%)	
Other	6 (3.4%)	1 (14.3%)	

*CLKT* Combined liver and kidney transplant, *SLKT* Sequential liver and kidney transplant, *IQR* Interquartile range, *BMI* Body mass index, *CNS* Central nervous system

Transplant outcomes are listed in Table 3. For liver outcomes, the incidence of rejection episodes between transplant and discharge was similar (12.9% in the CLKT group vs. 12.5% in the SLKT group, *p* = 0.898). Although not statistically significant (*p* = 0.371), no incidences of liver graft failure were observed in the SLKT group before discharge, while three patients (3.9%) developed graft failure in the CLKT group. Discharge laboratory values also demonstrated comparable outcomes in alanine aminotransferase, aspartate aminotransferase, alkaline phosphate, and albumin; while serum creatinine concentration was significantly different (*p* = 0.018).

**Table 3:** Transplant outcomes in CLKT and SLKT groups

	CLKT (***n*** = 181)	SLKT (***n*** = 20)	***P*** value
**Liver**			
Rejection episode	15 (12.9%)	1 (12.5%)	0.898
Graft failure	7 (3.9%)	0 (0.0%)	0.371
Cause of graft failure			
Primary graft failure	2 (33.3%)		
Vascular thrombosis	2 (33.3%)		
Acute rejection	1 (16.7%)		
Other	1 (16.7%)		
Recipient discharge labs (Median, IQR)			
AST (U/L)	44.0 (22.3,61.8)	35.5 (26.0,58.3)	0.912
ALT (U/L)	44.5 (21., 97.5)	38 (20, 88.5)	0.58
Alkaline phosphate (U/L)	250.5 (191, 501.8)	441.5 (254.0, 891.3)	0.083
Albumin (g/dL)	3.0 (2.43, 3.38)	2.9 (2.0, 3.6)	0.626
Serum creatinine (mg/dL)	1.4 (0.7, 2.4)	4.4 (3.7, 6.25)	0.018
Median days from transplant until discharge (IQR)	19 (9,34)	26 (14,33)	0.238
**Kidney**			
Rejection episode	5 (4.3%)	0 (0.0%)	0.505
Graft failure	15 (8.3%)	3 (15.0%)	0.321
Cause of graft failure			
Acute rejection	0 (0.0%)	1 (33.3%)	
Primary graft failure	3 (20.0%)	1 (33.3%)	
Graft thrombosis	2 (13.3%)	1 (33.3%)	
Infection	1 (6.7%)	0 (0.0%)	
Urological Complications	1 (6.7%)	0 (0.0%)	
Recurrent diseases	1 (6.7%)	0 (0.0%)	
Primary non-function	2 (13.3%)	0 (0.0%)	
Unknown	5 (33.3%)	0 (0.0%)	
Serum creatinine at discharge (mg/dL, median, IQR)	1.3 (0.7,2.3)	0.9 (0.45,1.4)	0.161
Median number of days from transplant until discharge (IQR)	19 (9,34)	10 (5,21)	0.009
Median interval between LT and KT (month, IQR)		10.5 (6.25, 24.25)	

*CLKT* Combined liver and kidney transplant, *SLKT* Sequential liver and kidney transplant, *IQR* Interquartile range, *BMI* Body mass index, *CNS* Central nervous system, *LT* Liver transplantation, *KT* Kidney transplantation, *AST* Aspartate aminotransferase, *ALT* Alanine aminotransferase

The incidence of rejection in KT between transplant and discharge was 5% in the CLKT group, while no rejection was observed in the SLKT group (*p* = 0.505). The incidence of graft failure was 8.3% in the CLKT group compared with 15% in the SLKT group (*p* = 0.321). The serum creatinine concentration at discharge was slightly lower in the SLKT group, although statistical significance was not reached (*p* = 0.161). The duration of hospitalization after transplantation in the SLKT group (median time = 10 days; IQR 5, 21) was much shorter than in the CLKT group (median time = 19 days, IQR 9, 34; *p* = 0.009). The median interval between LT and KT was 10.5 months (IQR 6.25, 24.25).

**Fig. 1:**
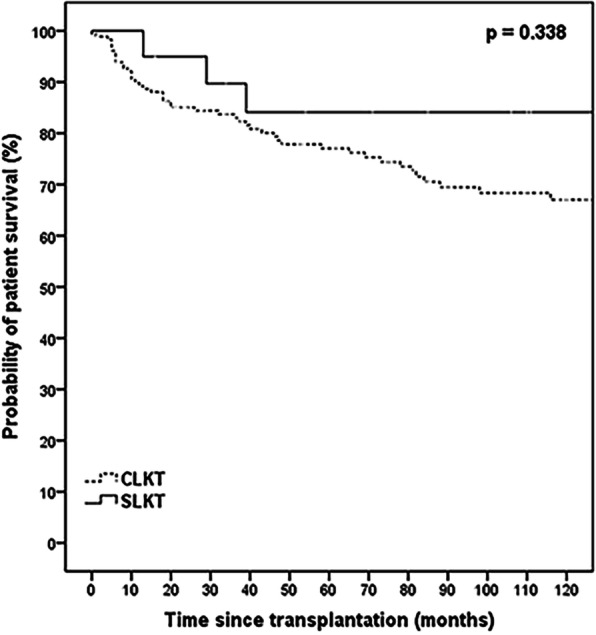
Kaplan–Meier curves for patient survival in the CLKT and SLKT groups. Recipients in CLKT had nonsignificant survival outcome compared with recipients in SLKT. Continuous line depicts recipients underwent SLKT and dashed line depicts recipients underwent CLKT

**Fig. 2:**
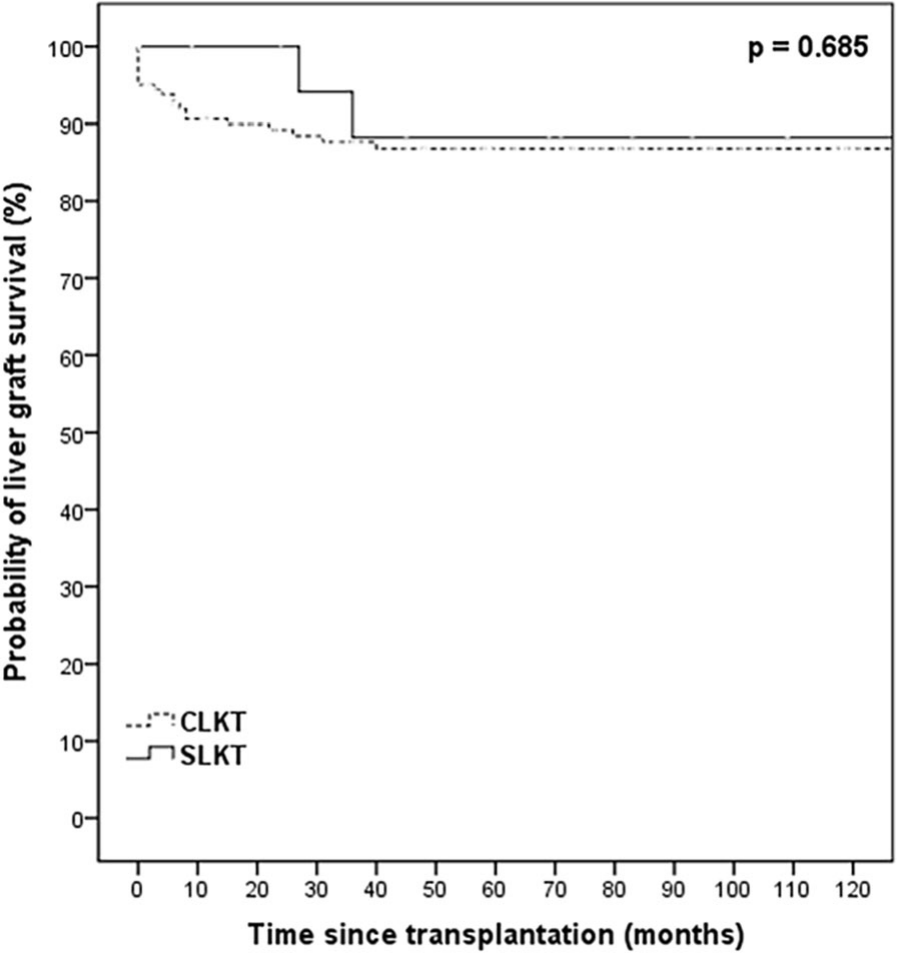
Kaplan–Meier curves for liver graft survival in the CLKT and SLKT groups. Recipients of liver graft in CLKT had comparable graft survival compared with recipients in SLKT. Continuous line depicts recipients underwent SLKT and dashed line depicts recipients underwent CLKT

**Fig. 3:**
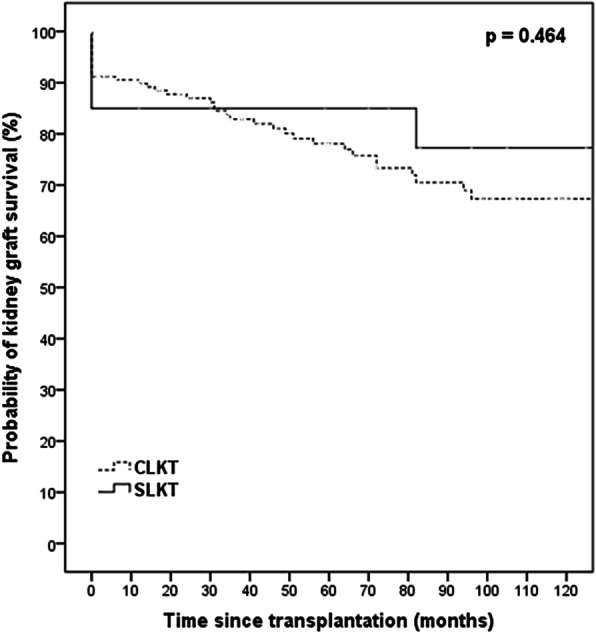
Kaplan–Meier curves for kidney graft survival in the CLKT and SLKT groups. Recipients of kidney graft in CLKT had comparable graft survival compared with recipients in SLKT. Continuous line depicts recipients underwent SLKT and dashed line depicts recipients underwent CLKT

Cumulative 1-year, 5-year, and 10-year patient survival rates were 89.2, 77.0, and 67.0% in the CLKT group; and 100, 84.1, and 84.1% in the SLKT, respectively (Fig. 1, *p* = 0.338). Although not statistically significant, the SLKT group presented with better outcomes. The cumulative 5-year liver graft survival rate was 86.8% in the CLKT group, and 88.2% in the SLKT group (Fig. 2, *p* = 0.685). The cumulative 5-year kidney graft survival rate was 78.1% in the CLKT group and 85.0% in the SLKT group (Fig. 3, *p* = 0.464).

## Discussion

LKT is the only curable treatment for PH because it solves both hepatic enzyme disorder and renal dysfunction, in which the CLKT is a globally implemented LKT procedure with abundant experience. However, SLKT seems to be a much more feasible procedure because it divides the LKT into two steps: LT followed by KT. The application of post-transplant intensive dialysis could help liver graft to be free from damage caused by a high degree of system oxalate deposition. This procedure has succeeded in living donor transplants with encouraging outcomes [14]. Outcomes of LKT in patients with PH1 have improved over the decades, particularly in CLKT [15]; however, published data investigating SLKT is limited.

Some clinicians have highlighted that SLKT suits patients with long-term renal replace treatment or patients suffering with systemic oxalosis, while CLKT suits patients with a short dialysis period or without systemic oxalosis [14]. Our study demonstrated that the SLKT group had similar preoperative renal function at LT compared with the CLKT group, and the long-term outcomes of patient survival and graft loss were encouraging in the SLKT group when compared with the CLKT group. The cumulative 1-year, 5-year, and 10-year patient survival rates were 89.2, 77.0, 67.0% in the CLKT group, and 100, 84.1, 84.1% in the SLKT group. The cumulative 5-year liver graft survival rate was 86.8% in the CLKT group and 88.2% in the SLKT group, and the cumulative 5-year kidney graft survival rate was 78.1% in the CLKT group and 85.0% in the SLKT group. However, these two groups of patients seemed to be at a different level of pretransplant renal condition demonstrated by the higher proportion of patients under dialysis and longer pretransplant duration (17.0 months in CLKT (IQR 7.0, 22.0) vs. 4.0 months in SLKT (IQR 0.0, 12.3), *p* < 0.001) in the CLKT group compared with the SLKT group. Then we simply compared pretransplant renal function between 2 groups by serum creatinine because of lacking of GFR data before LT in SLKT groups, however, the serum creatinine showed no significant difference (*p* = 0.312).

Donor condition should also take into consideration. Most CLKT procedures included in the present study utilized organs from single deceased donors. For SLKT, one of the organs is often procured from a living donor and the other from a deceased donor [16–18]. We also demonstrate this organ allocation preference in our patient’s demographic statistics analysis: the CLKT group utilized 98.9% of organs from a single deceased donor, while the SLKT group utilized 75% of livers from deceased donors and only 35% of kidneys from deceased donors.

Research suggests that liver grafts have a protective effect on kidney grafts when procured from the same donor and when transplanted simultaneously, which translates to a low rate of acute rejection and improved kidney graft survival when compared with isolated KT or LT [19, 20]. human leukocyte antigen (HLA) are one the suggested mechanisms behind the immunoprotective phenomenon [21, 22]. HLA antigens produced by the transplanted liver allograft could neutralize circulating alloantibodies. Soluble HLA antigens delivered by the liver allograft could inhibit natural killer and cytotoxic T cells. Kupfer cells and biliary epithelial cells from the liver allograft also demonstrate an ability to clear circulating antigens [23, 24]. Our study may support this hypothesis through the observation that the SLKT group acquired significantly lower mismatch scores when compared with the CLKT group both in liver grafts (*p* = 0.003) and renal grafts (*p* < 0.001) but reached a comparable long-term outcome either in patient survival or graft loss.

Even though the deficiency in specific hepatic enzymes could be solved by liver allograft, renal allograft may accumulate oxalate deposits while mobilizing and eliminating systemic oxalate accumulations [18, 25]. Therefore, SLKT seems to be more reasonable than CLKT for patients with severe plasma oxalate accumulation because it decrease the plasma oxalate level below the saturation level by LT and intensified dialysis protocol to create a better kidney graft survival condition before KT. Studies have reported that patients undergoing SLKT who underwent an early LT could acquire decreased perioperative morbidity and therefore more choices for kidney allocation (either a deceased or a living donor) compared with those only undergoing dialysis [26]. However, the optimal interval between LT and KT is unknown. In 2017 a United Network for Organ Sharing (UNOS) allocation policy for CLKT was implemented, which defines the medical eligibility criteria for CLKT and provides a safety net by assigning priority for renal allograft allocation to LT recipients with ESKD within 1 year of LT [27, 28]. In the present study, the median interval between KT and LT was 10.5 months (IQR 6.25, 24.25) and the long-term outcome somewhat proved the validity of these allocation policies.

The present study is limited by its retrospective design. First, the diagnosis of PH is obscure owing to lack of genetic diagnosis data, so the subtype of PH could not be analyzed. Second, the details of patient status, such as oxalate concentration and glomerular filtration rate were not mentioned; we could only simply assess renal function using the recipient serum creatinine concentration and pretransplant dialysis condition. Third, our result may have overestimated the outcome of SLKT because the cases we retrieved from the database were with definite LKT records, making the cases who intended to receive SLKT but failed by the failure of LT were excluded. Furthermore, the sample size was relatively small; a few cases of post-transplant death and graft loss were recorded, making it hard to identify risk factors and perform multivariate assessment.

In summary, PH is a rare inherited disease that is associated with substantial metabolic disorders and high mortality if it is not treated effectively, and LKT is critical for the majority of patients with PH. Our results may suggest that SLKT could be a viable alternative treatment to CLKT in specific patients with limited conditions including early LT procedure and appropriate donor resource. Based on this suggestion, further exploration about the SLKT such as pretransplant condition, donor allocation and interval between the transplantations is still required.

## Conclusions

In conclusion, our study demonstrated that the SLKT seems to be an alternative option with strict condition for CLKT, further exploration about the SLKT is still required.

## Data Availability

The data that support the findings of this study are available from the Scientific Registry of Transplant Recipients database, but restrictions apply to the availability of these data, which were used under license for the current study, and so are not publicly available. Data are however available from the authors upon reasonable request and with permission of the Scientific Registry of Transplant Recipients database.

## Abbreviations

CLKT: Combined liver and kidney transplant
ESKD: End-stage kidney disease;
HLA: Human leukocyte antigen
KT: Kidney transplant
LKT: Liver-kidney transplant
LT: Liver transplant
PH: Primary hyperoxaluria
PH1: Primary hyperoxaluria type 1
SLKT: Sequential liver and kidney transplant
